# Peripheral refraction of myopic eyes with spectacle lenses correction and lens free emmetropes during accommodation

**DOI:** 10.1186/s40662-021-00267-x

**Published:** 2021-12-01

**Authors:** António Queirós, Alejandro Cerviño, José Manuel González-Méijome

**Affiliations:** 1 Clinical & Experimental Optometry Research Lab (CEORLab), Center of Physics (Optometry), University of Minho, Campus de Gualtar, 4710-057 Braga, Portugal; 2 Optometry Research Group, Department of Optics and Optometry and Vision Sciences, University of Valencia, Valencia, Spain

**Keywords:** Off-axis refraction, Refractive error, Accommodation, Myopia progression

## Abstract

**Purpose:**

To measure axial and off-axis refraction patterns in myopic eyes with spectacle lenses correction and lens free emmetropes in young healthy subjects at different target distances from 2.00 m (0.50 D) to 0.20 m (5.00 D) in terms of sphere, astigmatism, and spherical equivalent refraction.

**Methods:**

Refraction was measured at the center, 20 and 40 degrees from the line of sight both nasally and temporally in 15 emmetropic and 25 myopic young healthy subjects with an open field, binocular, infrared autorefractor (Grand Seiko WAM-5500, Hiroshima, Japan). Fixation target was a Maltese cross set at 2.00, 0.50, 0.33 and 0.20 m from the corneal plane. Changes in off-axis refraction with accommodation level were normalized with respect to distance axial values and compared between myopic eyes with spectacle lenses correction and lens free emmetropes.

**Results:**

Off-axis refraction in myopic eyes with spectacle lenses correction was significantly more myopic in the temporal retina compared to lens free emmetropes except for the closest target distance. Relative off-axis refractive error changed significantly with accommodation when compared to axial refraction particularly in the myopic group. This change in the negative direction was due to changes in the spherical component of refraction that became more myopic relative to the center at the 0.20 m distance as the J0 component of astigmatism was significantly reduced in both emmetropes and myopes for the closest target.

**Conclusion:**

Accommodation to very near targets (up to 0.20 m) makes the off-axis refraction of myopes wearing their spectacle correction similar to that of lens free emmetropes. A significant reduction in off-axis astigmatism was also observed for the 0.20 m distance.

## Introduction

Despite increasing knowledge on the developmental aspects of the visual system, one of the most enigmatic areas in visual development is to understand the reason why a given eye could become myopic and why myopia could progress to achieve pathological levels. Nowadays, this is a public health concern, as more than 25% of the population in developed countries [1] and perhaps as much as 70% in some Asian regions [2, 3] suffer myopia.

During the last decade, several developments in the field of optometry have provided strong evidence of several environmental and genetic characteristics, as well as parental history of myopia, as factors potentially involved in the determination of the refractive status of the eye [4–6]. Near work has been considered one of the factors for explaining the increasing prevalence of myopia world-wide [7–9]. It seems that accommodative lag during sustained near work could be involved in myopia progression, particularly in patients with near esophoria [10–12], although there is some controversy in the association with the progression of myopia [13, 14].

Several studies have proposed that the pattern of peripheral refraction could also be involved in the progression of refractive error. This assumption comes from separate evidence reported in different studies. First, it has been demonstrated that myopic patients have comparatively less myopia in the peripheral retina [15], compared to the central refraction, leading to a potential peripheral hyperopia when axial refraction is compensated with eyeglasses or single vision contact lenses. This might be exacerbated by the fact that myopic eyes have a less oblate or prolate posterior retinal shape compared to the predominantly oblate shape among emmetropic and hyperopic eyes [16, 17] due to the different patterns of growth in the axial and equatorial dimensions of those eyes [17, 18]. Second, some optical treatments that invert the profile of peripheral refraction in myopic eyes from relative peripheral hyperopia to more myopic peripheral refraction [19–21] due to changes induced in the corneal surface [22] have been cited as the main reason behind the lower ocular growth reported in either separate report of cases [23] or controlled trials in Spain [24], Hong Kong SAR, China [25], USA [26], and Japan [27]. Third, to confirm the hypothesis that peripheral refraction could interfere with the ocular growth pattern of myopic eyes, studies conducted by Smith and colleagues in animal models showed that visual experience in the peripheral retina could interfere with this process [28]. Furthermore, clinical studies have also shown that the peripheral refractive profile along the horizontal meridian could also play a role on the onset and progression of myopia in children with eyes having less myopic or hyperopic peripheral refractive patterns [29].

Considering all the aforementioned factors, we hypothesize that any change in peripheral refraction in the hyperopic direction with accommodation in myopic eyes could lead to an increased risk of myopia progression in predisposed eyes due to their commonly recognized pattern of peripheral hyperopic refraction compared to axial refraction. This issue has been investigated by Calver et al. with no apparent effect of accommodation on peripheral refractive patterns [30]. However, the closer target in near vision conditions was set at 0.40 m. Subsequently, shorter distances (0.30 m) in myopes were analyzed by Whatham et al. [31].

In this report, we evaluated if using closer targets at 0.33 m and 0.20 m could cause some change in the peripheral refractive pattern in myopic eyes with spectacle lenses correction as compared to lens free emmetropic eyes that could justify a higher risk of myopia progression under highly accommodative stressful conditions. Previous studies had not investigated the effect of such close stimulus (higher accommodative demands) on peripheral refraction.

## Material and methods

Axial and off-axis refractive error was measured in 15 lens free emmetropic and 25 myopic eyes with spectacle lenses correction at several fixation distances. Only the right eye of each patient was measured. Demographic details about the patients enrolled are presented in Table 1. Good ocular health of all the volunteer subjects was assessed by optometric examination prior to enrolment in the study. None of the patients had any active ocular pathology or previous surgery nor had astigmatism greater than 0.75 D. All the participants read and signed an informed consent prior to examination. The protocol was approved by the Ethics Subcommittee for Life and Health Sciences of the University of Minho and followed the guidelines of the Declaration of Helsinki. Measurements were taken at the Clinical and Experimental Optometry Research Lab under controlled room illumination to ensure a non-pharmacologically induced pupil size that warrants proper acquisition of refraction even at oblique viewing.

**Table 1:** Demographic data for myopic and emmetropic patients

	Myopes (n = 25)	Emmetropes (n = 15)
Gender	4 male/21 female	2 male/13 female
Age (years)	22.27 ± 4.45	22.12 ± 2.95
Sphere (D)	− 2.16 ± 1.28	0.02 ± 0.10
Cylinder (D)	− 0.51 ± 0.66	− 0.07 ± 0.27
Spherical equivalent (D)	− 2.42 ± 1.36	− 0.02 ± 0.17

Measurement of peripheral refraction was done monocularly using an infrared binocular open-field autorefractometer (Grand Seiko Auto Ref/Keratometer WAM-5500, Grand Seiko Co. Ltd., Hiroshima, Japan) [32, 33]. Contralateral eye was occluded during measurement acquisition. Fixation target is a Maltese cross positioned at the center and at 20 and 40 degrees from the line of sight in straight ahead gaze, both nasally and temporally from the visual axis. Target was set at 2.00 m, 0.50 m, 0.33 m, and 0.20 m from the examined eye for each eccentricity. Subjects were not cyclopleged prior to data acquisition. Mean of five consecutive measurements were obtained for each position and target distance. Refractive data were normalized by subtracting the refractive value obtained from each patient for the central point to all the remaining values including those from different distances and different eccentricities. This does not have any impact on the shape of the refractive profiles, but only shifts them for easier comparison between myopic and emmetropic eyes at different fixation distances. The intraocular pressure was verified with a noncontact tonometer before measurements acquisitions to rule out the influence on accommodation [34, 35].

Myopic patients were wearing their spectacle correction to ensure the appropriate level of accommodation. Although this fact could affect peripheral astigmatism, results from the authors have shown that this is not the case when using the Grand Seiko Auto-refractor as previously reported in patients wearing fogging lenses [36, 37]. Turning the eye or the head could also be a source of variability when assessing peripheral refraction. In our study however, the subjects were asked to maintain the head still and only rotate the eyes for fixation of peripheral points to minimize the effect of the lens, following the protocol previously described by Calver et al.[30]. This approach is also supported by the fact, reported by Radhakrishnan and Charman, that turning the head or the eye for the measurements of peripheral refraction would not render significantly different results [38].

### Statistical analysis

The refractive error obtained was converted from sphero-cylindrical notation to vectorial components spherical equivalent (SE = Sphere + Cylinder/2), J0 and J45 according to Fourier analysis, prior to statistical analysis using the equations derived by Thibos et al. [39].

Sphere and spherical equivalent were analyzed for each target distance and eccentricity. This might be considered somewhat redundant because spherical equivalent also includes the spherical component. However, as the accommodative response of the eye changes essentially the spherical component of refraction, we are particularly interested in analyzing this separately without the contribution of astigmatism. Statistical comparisons were performed to evaluate the presence of differences in relative off-axis refraction for different refractive targets between myopes and emmetropes and between accommodative level using Mann-Whitney U test for non-parametric data and independent sample t-test for normally distributed data to assess differences between myopes and emmetropes at each given location and fixation target. For comparisons between refractive targets within each refractive group (myopic or emmetropic) or between central and peripheral eccentricities for a given vergence, Kruskal-Wallis and one-way ANOVA with Bonferroni post-hoc correction tests were used for non-parametric and parametric data, respectively to evaluate differences in refraction for each retinal location as a function of the fixation distance. Finally, paired samples t-test and Wilcoxon signed ranks test were used to compare the peripheral refractive data to the central one for each given fixation distance to assess significance of relative peripheral refractive error. Plots of peripheral refraction according to eccentricity and accommodation level were made for each refractive group. All data have been normalized by subtracting the central value at 2.00 m to all the remaining values to highlight the different profiles at each given accommodative level. The SPSS Statistical Package for Social Sciences (v.22.0, SPSS Inc, Chicago, IL) was used for statistical analyses.

## Results

### Effect of ametropia (emmetropic vs myopic eyes)

**Fig. 1:**
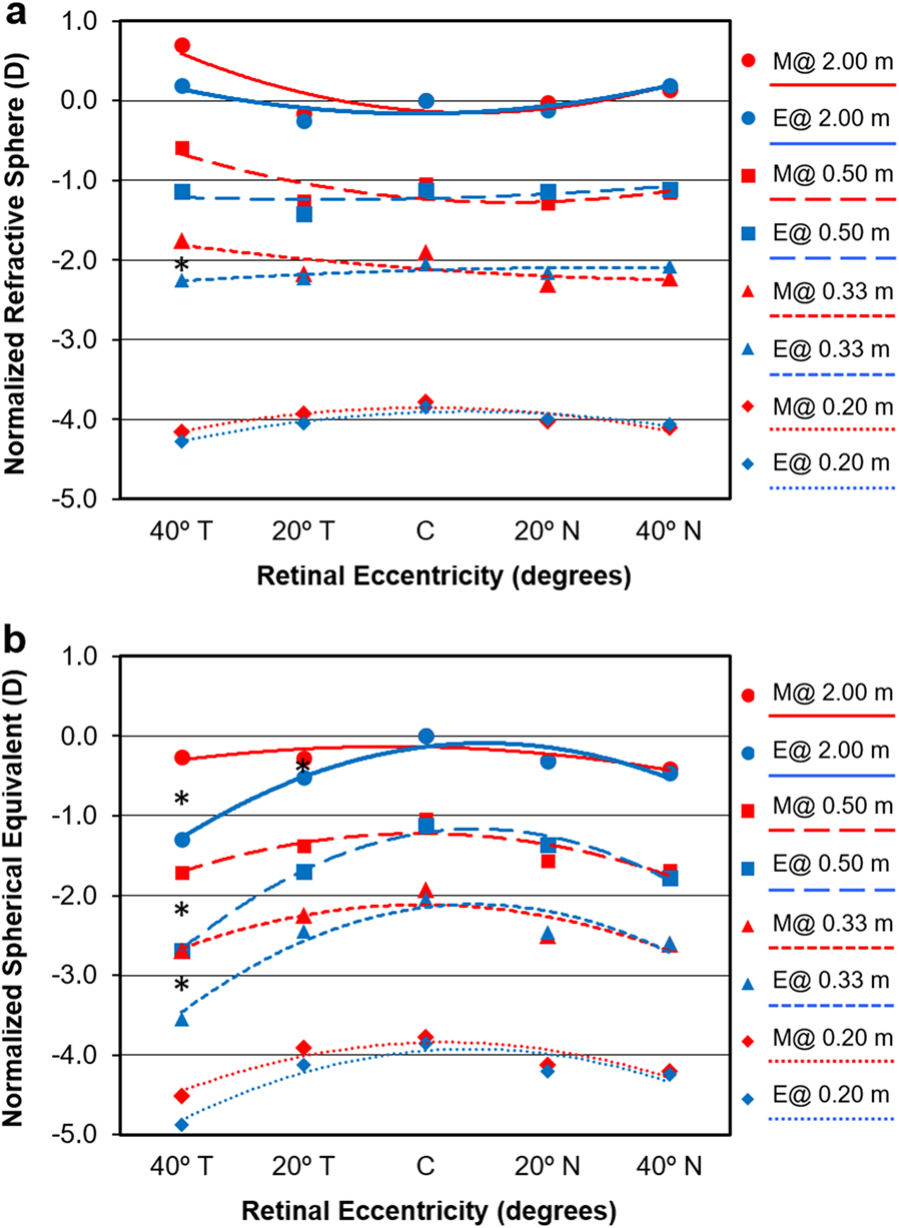
Mean profiles of central and off-axis refraction in terms of sphere (**a**) and spherical equivalent (**b**) for myopic eyes with spectacle lenses (M, red markers and lines) and lens free emmetropic (E, blue markers and lines) patients at each fixation distance. The axial refractive sphere at 2.00 m was subtracted to all remaining values to bring the 2.00 m curve to “zero” at center. Error bars have been omitted for clarity. *Statistically significant differences for vergence between myopes and emmetropes

Figure 1a shows the refractive profiles for myopic eyes with spectacle lenses correction and lens free emmetropes at different eccentricity for each accommodating target. Myopic eyes with spectacle lenses correction show a temporal refractive error that is less negative than lens free emmetropes although these differences were only statistically significant for the 0.33 m distance with myopes spectacle lenses correction showing more hyperopic temporal peripheral refraction than lens free emmetropes. Interestingly, refractive profiles for both groups at 0.20 m are almost identical. Figure 1b shows the results for spherical equivalent refraction, showing statistically significant differences for all distances at the 40º temporal location except for 0.20 m where again the refractive profiles of both groups are quite similar.

**Fig. 2:**
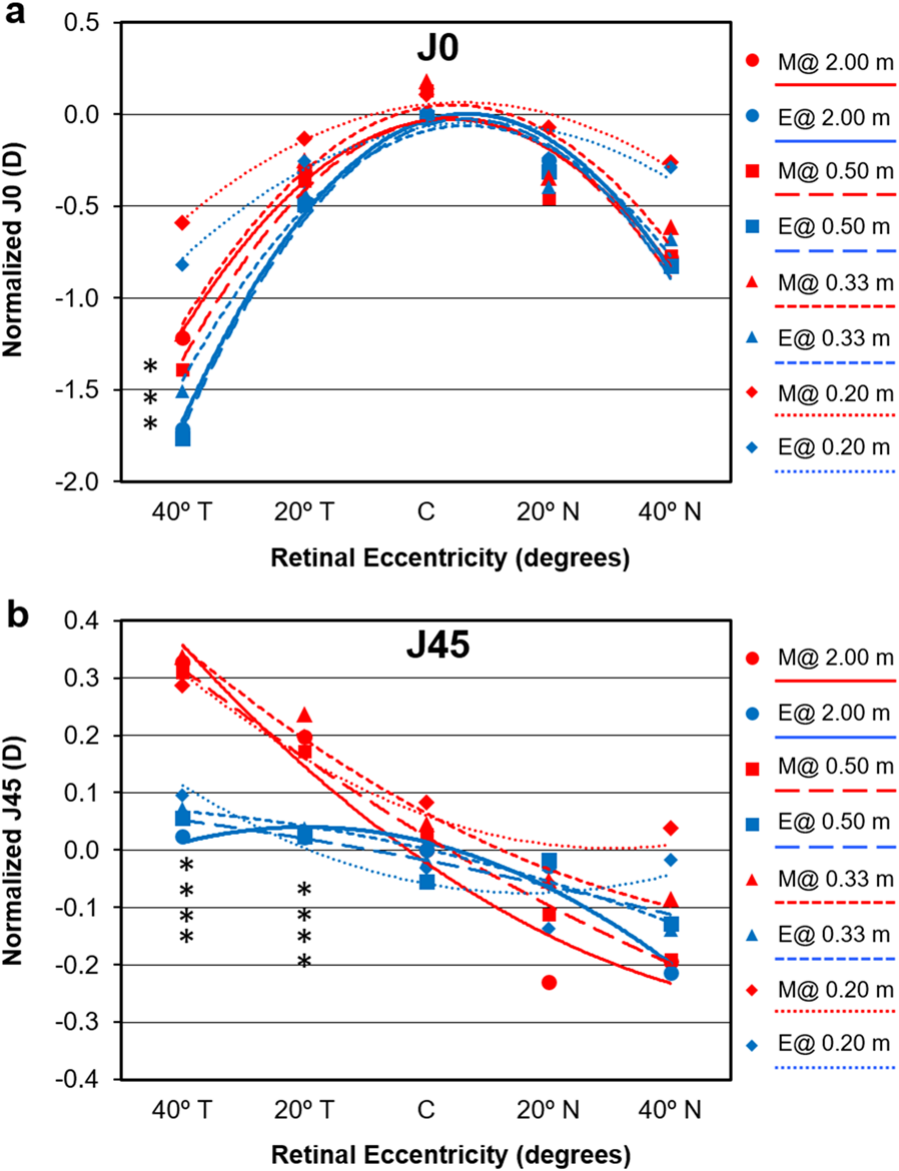
Mean profiles of central and off-axis refraction in terms of astigmatic vector components J0 (**a**) and J45 (**b**) myopic eyes with spectacle lenses (M, red markers and lines) and lens free emmetropic (E, blue markers and lines) patients at each fixation distance. The axial spherical equivalent at 2.00 m was subtracted to all remaining values to bring the 2.00 m curve to “zero” at center. Error bars have been omitted for clarity. *Statistically significant differences for vergence between myopes and emmetropes

Figure 2 shows the refractive profiles in terms of astigmatic components J0 and J45. Differences in J0 at 40º temporal were present between myopic eyes with spectacle lenses correction and lens free emmetropes (*P* ≤ 0.022) except for the 0.20 m fixation distance (*P* = 0.054, Mann-Whitney U test). There were statistically significant differences in J45 between myopic eyes with spectacle lenses correction and lens free emmetropes for all fixation distances at 20° and 40° temporal (*P* ≤ 0.012).

### Relative peripheral refractive error (compared to axial refraction)

This analysis renders interesting results that are shown qualitatively in Figs. 1 and 2. By comparing each peripheral point to the central refraction for a given group and for a given accommodative target, differences are evident between myopic eyes with spectacle lenses correction and lens free emmetropes. The spherical component of myopes was not significantly different in the periphery *vs.* the center except for 40º temporal at 2 m where the refraction is more hyperopic (*P* = 0.047, paired samples t-test). Conversely, in the emmetropic group, several peripheral points, particularly in the temporal field are significantly more myopic than the central refraction (*P* ≤ 0.039, paired samples t-test). Concerning spherical equivalent, while myopes with spectacle lenses correction only showed significantly more myopic values at the periphery than at the center for the 0.33 and 0.50 m distance fixation both nasally and temporally (*P* = 0.041), all eccentric points in the lens free emmetropic group showed a significantly more myopic refraction compared to center at every fixation distance (*P* ≤ 0.006).

### Effect of accommodative demand (0.50 to 5.00 D)

**Fig. 3:**
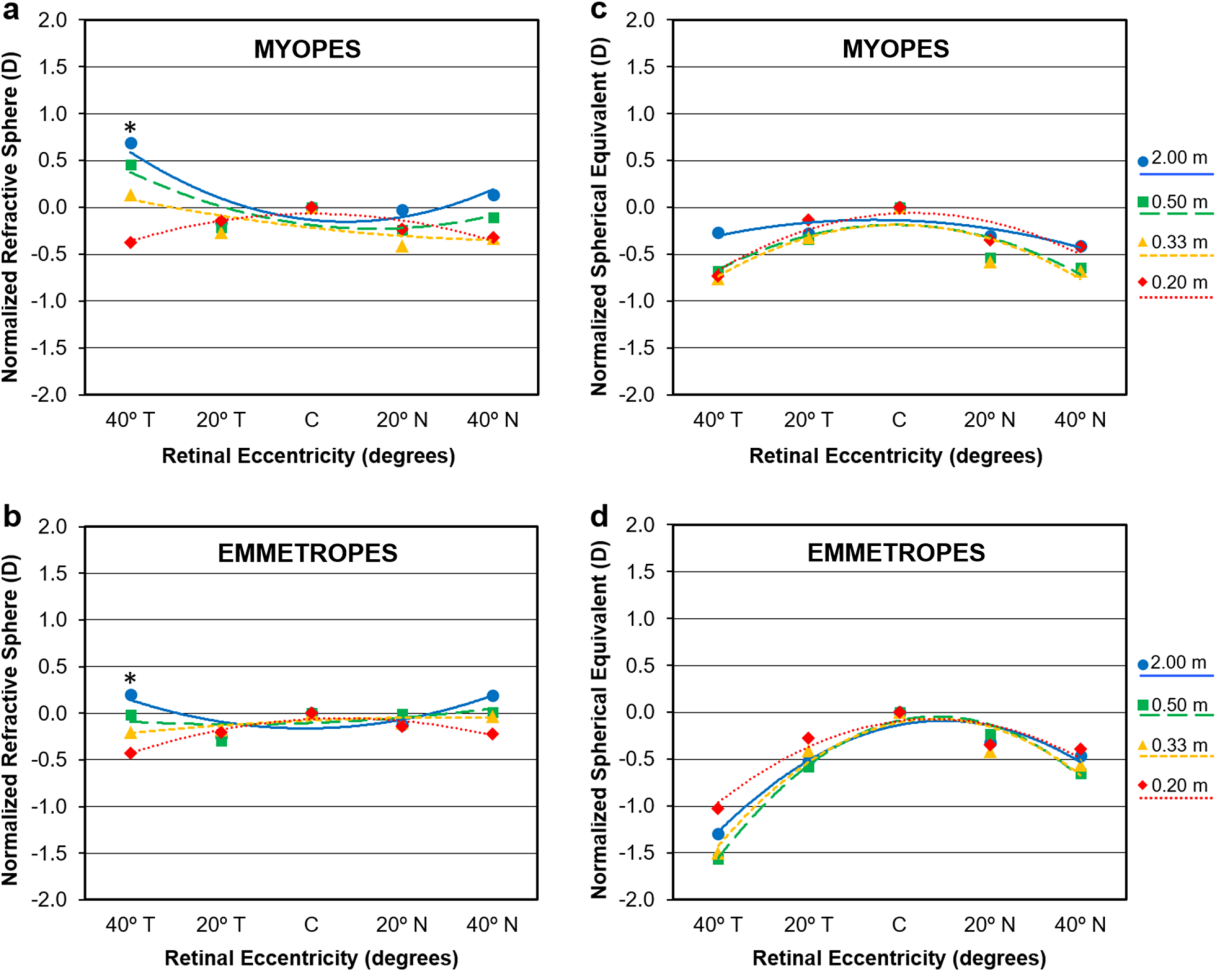
Mean profiles of central and off-axis refraction in terms of sphere (**a** and **b**) and spherical equivalent (**c** and **d**) for myopic eyes with spectacle lenses and lens-free emmetropic patients at each fixation distance. All data have been normalized by subtracting the central value at each fixation distance to the remaining respective off-axis values to highlight the different profiles at each given accommodative level. Error bars have been omitted for clarity. *Statistically significant differences for vergence

Figure 3 represents the comparison of the refractive profiles where the central value at each fixation distance was subtracted to the peripheral ones to shift the curve to “zero” at center. This makes any comparison among fixation distances much easier. Figure 3a and b show that the spherical refraction at 40° T is significantly different for the different distances in myopes with spectacle lenses correction and lens free emmetropes (*P* = 0.030 and *P* = 0.005, respectively) using one-way ANOVA with Bonferroni post-hoc correction. Figure 3c and d display the same effect for spherical equivalent refraction. The results previously revealed for the spherical component are hidden by the introduction of the astigmatic component. Consequently, no statistically significant differences were observed among accommodative demand either for myopic eyes with spectacle lenses correction or lens free emmetropes.

**Fig. 4:**
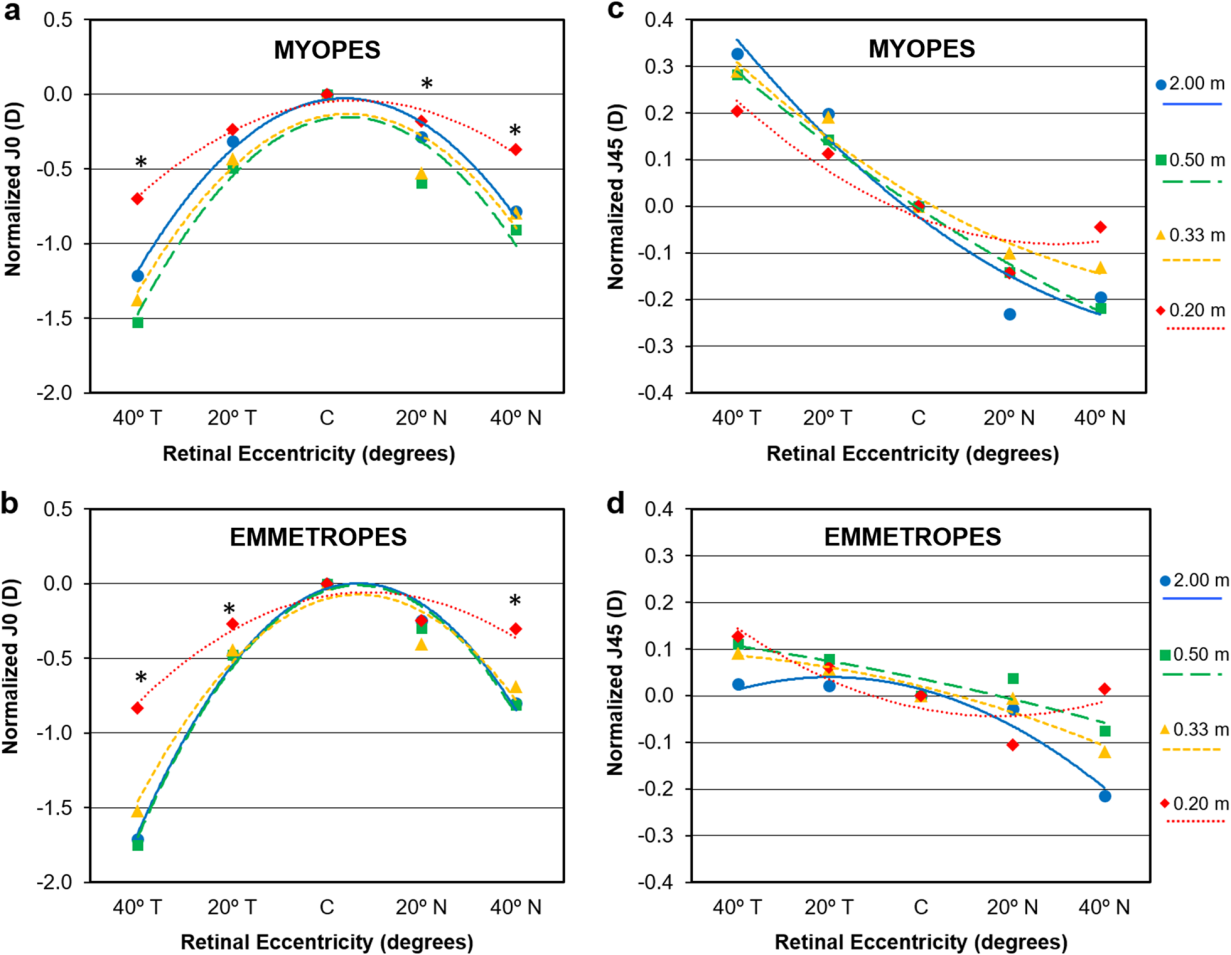
Mean profiles of central and off-axis refraction in terms of astigmatic vector components J0 (**a** and **b**) and J45 (**c** and **d**) for myopic eyes with spectacle lenses and lens-free emmetropic patients at each fixation distance. All data have been normalized by subtracting the central value at 2.00 m to all the remaining values to highlight the different profiles at each given accommodative level. Error bars have been omitted for clarity. *Statistically significant differences for vergence

Figure 4a and b show how the J0 astigmatic component reduces in both myopic and emmetropic groups for the closest accommodative target (5.00 D at 0.20 m). Changes are less evident in the J45 component although a significant change in pattern is again observed for the 0.20 m fixation distance.

## Discussion

The effects of accommodation in peripheral refraction have been the subject of research in different studies conducted over the last few years [12, 30, 31, 40–44]. However, different studies arrived to slightly different conclusions. Except for Lundstrom et al. who performed the measurements with a laboratory Hartmann-Shack wavefront sensor, all the others used the principle of autorefraction.

Lundstrom et al. measured peripheral refractions at accommodative demands of 0.50 and 4.00 D and found that myopes did not show a consistent change in peripheral refractive profile while emmetropes became relatively more myopic in the periphery with accommodation [43]. Mathur et al. showed that emmetropes maintained their relative peripheral myopia at two accommodative demands, 0.30 and 4.00 D [44], while Whatham et al. examined peripheral refractions in myopes at three levels of accommodation up to 3.33 D and found that myopes display hyperopic shifts in the near periphery with accommodation but their far peripheral refraction can shift in the myopic direction [31].

Our results agree with those reported by Calver et al. [30] of similar changes (at distance and 40 cm) in peripheral refraction for both myopes and emmetropes with accommodation. Hence, these results do not support the hypothesis of changes in peripheral refraction during near vision tasks as a precursor of myopia development even for closer targets up to 20 cm (5.00 D of vergence). Indeed, no previous study had analyzed the changes in peripheral refraction for such closer vergences. These results also agree with previous findings of differences in peripheral refraction between myopes and emmetropes for distance vision. While relative refractive shift in the nasal retina is similar for both refractive groups, refractive shift in the temporal retina is not, yielding significantly more myopic refractive values for emmetropic subjects than myopic subjects.

Our results also show that peripheral refraction during high accommodative levels is not significantly different between emmetropes and myopes. The differences observed in mean sphere equivalent for distance vision for temporal eccentricities are maintained for intermediate and near vision up to 3.00 D, however, those differences decrease to become non-significant for higher levels of accommodation (around 4.00 D in the present study). Those differences in peripheral mean sphere equivalent between both groups are attributable to peripheral astigmatism since results failed to show significant differences between myopes and emmetropes in the spherical component for any accommodative level studied.

Sample size could be considered one limitation of the study. However, sample size calculation determined that to detect differences of 0.50 D between central and peripheral refraction within each group (at different eccentricities), 15 patients would be needed to have a statistical power of 80% for a significance *P* value of 0.05. To detect differences in peripheral refraction among different groups (myopes *vs*. emmetropes), 14 patients would be necessary in each group considering the same statistical power and significance value. Despite the limitations imposed by the relatively small sample size, this sample warrants confidence on the results obtained. Another limitation of this study is that we only evaluated lower order aberrations, defocus, and astigmatism. However, at present, the higher and most consistent changes in peripheral refraction are in spherical and astigmatic defocus, and thus were the terms of refraction investigated. However, the fact that higher order aberrations also change in peripheral viewing [45, 46] and with accommodation [47], future studies to provide a more comprehensive insight on the changes with accommodation in peripheral vision are warranted. Another limitation of this study is the value of myopic subjects. However, it would be interesting in the future to perform analyses of peripheral refraction with accommodation for myopes greater than 6.00 D. Therefore, axial myopia which is associated with a decrease in the sclera, posterior choroid and retinal pigment epithelium [48], shows that this effect has no influence during orthokeratology on the substantial increment of peripheral myopic defocus [49].

Differences in peripheral refraction in the temporal retina between myopes and emmetropes observed for distance vision are attributable to peripheral astigmatism and maintained for intermediate and near tasks up to 0.33 m while disappearing for closer working distances.

In summary, the analysis of the data allows us to conclude that results reported here agree with those reported by Calver et al. [30] of similar changes in peripheral refraction for both myopes and emmetropes with accommodation. Hence, these results do not support the hypothesis of changes in peripheral refraction during near vision tasks as a precursor of myopia development. Rather than that, myopic eyes seem to behave similarly to emmetropic eyes for the closer accommodative targets. Results also agree with previous findings of differences in peripheral refraction between myopes and emmetropes for distance vision. While relative refractive shift in the nasal retina is similar for both refractive groups, refractive shift in the temporal retina is not, yielding significantly more myopic refractive values for emmetropic subjects than myopic subjects.

## Conclusion

Peripheral refraction during high accommodative levels is not significantly different between emmetropes and myopes. The differences observed in spherical equivalent refraction for distance vision for temporal eccentricities are maintained for intermediate and near vision up to 3.00 D, however those differences decrease to become insignificant for higher levels of accommodation (around 4.00 D in this study). Those differences in peripheral spherical equivalent refraction between both groups are attributable to peripheral astigmatism since results failed to show significant differences between myopic eyes with spectacle lenses and lens free emmetropes in the spherical component for any accommodative level studied except for 40 degrees temporally for the 3.00 D stimulus.

## Data Availability

Not applicable.
